# Benefits of different intensity of aerobic exercise in modulating body composition among obese young adults: a pilot randomized controlled trial

**DOI:** 10.1186/s12955-017-0743-4

**Published:** 2017-08-24

**Authors:** Chih-Hui Chiu, Ming-Chen Ko, Long-Shan Wu, Ding-Peng Yeh, Nai-Wen Kan, Po-Fu Lee, Jenn-Woei Hsieh, Ching-Yu Tseng, Chien-Chang Ho

**Affiliations:** 1 Department of Exercise Health Science, National Taiwan University of Sport, Taichung City, 40404 Taiwan; 2 Department of Physical Education, Fu Jen Catholic University, New Taipei City, 24205 Taiwan; 3 Graduate Institute of Sports Training, University of Taipei, Taipei City, 11153 Taiwan; 4 Center for General Education, Taipei Medical University, Taipei City, 11031 Taiwan; 5 Graduate Institute of Sport Coaching Science, Chinese Culture University, Taipei City, 11114 Taiwan

**Keywords:** Obesity, Aerobic exercise, Exercise intensity, Body composition

## Abstract

**Background:**

The aim of present study was to compare the effects of different aerobic exercise intensities and energy expenditures on the body composition of sedentary obese college students in Taiwan.

**Methods:**

Forty-eight obese participants [body mass index (BMI) ≥ 27 kg/m^2^, age 18–26 years] were randomized into four equal groups (*n* = 12): light-intensity training group (LITG), 40%–50% heart rate reserve (HRR); middle-intensity training group (MITG), 50%–70% HRR; high-intensity training group (HITG), 70%–80% HRR; and control group (CG). The aerobic exercise training program was conducted for 60 min per day on a treadmill 3 days per week for 12 weeks. All participant anthropometric data, blood biochemical parameters, and health-related physical fitness components were measured at baseline and after 12 weeks.

**Results:**

At baseline, the anthropometric indices did not differ significantly among the four groups (*p* > 0.05). After 12-week exercise intervention, the HITG and MITG had significantly more changes in body weight, waist circumference (WC), waist-to-hip ratio (WHR), and waist-to-height ratio (WHtR) than the LITG. The changes in BMI and body fat percentage differed among all four groups (*p* < 0.05).

**Conclusions:**

A 12-week high-intensity exercise intervention with high energy expenditure can considerably reduce body weight, body fat, WC, WHR, and WHtR, whereas a light-intensity exercise intervention can significantly reduce body weight and body fat.

**Trial registration:**

Current Controlled Trials TPECTR09831410900, registered on 24^th^ Dec 2009.

## Background

The percentage of the population that is obese increased in both the developed and developing countries [1]. Obesity is defined as a condition of abnormal or excessive body fat (i.e., the accumulation of adipose tissue) to the extent that health may be impaired [2]. Studies have demonstrated that obesity is linked to higher risk of developing various chronic diseases, such as cardiovascular disease, type 2 diabetes mellitus, and cancers [3, 4]. According to the clinical standards for defining obesity proposed by Taiwan’s Ministry of Health and Welfare (MOHW), children and adolescents are defined as obese based on their body mass index (BMI) over age- and sex-specific 95th percentile values; by contrast, adults with a BMI ≥ 27 kg/m^2^ are defined as being obese.Nevertheless, the standards for “obesity” in adults are also diverse among different countries. For example, the World Health Organization (WHO)-Asian standard for obesity in adults is a BMI ≥ 25 kg/m^2^, in contrast to the obesity standard set by the MOHW in Taiwan [5]. The MOHW’s standard is the more frequently used anthropometric measure to evaluate the degree of obesity among Taiwanese adults. Based on data from the three waves of the Nutrition and Health Surveys in Taiwan (NAHSIT), which occurred in 1993–1966, 2005–2008, and 2013–2014, Chang et al. [6] noted that the prevalence of adult obesity has drastically increased in recent decades from 11.8% to 17.9% to 22.1%, respectively. Two earlier surveys were conducted using multiple stage sampling among Taiwanese college students, wherein the prevalence of overweight and obesity was 31.4% in men and 16.4% in women [7, 8]. Weight loss is crucial for improving health and reducing body fat [9]. Thus, considering the public health, weight loss is effective for reducing chronic disease risk [10].

Aerobic exercise increases peak oxygen consumption (VO_2_ peak), which is closely correlated with total body fat percentage (BF%); aerobic exercise is also a powerful strategy for weight loss, particularly body fat loss [11, 12]. When designing a suitable weight loss program, exercise duration and intensity are generally manipulated. Moderate aerobic exercise for at least 150 min per week may improve risk factors for metabolic syndrome like body composition, insulin resistance and glycated haemoglobin (HbA1c) [13]. American College of Sports Medicine suggested that long-term moderate aerobic exercise for >150 or 200–300 min per week can significantly reduce body weight when the diet is not controlled [10]. However, when exercise intensities differ, exercise expenditure is not the only factor responsible for weight loss [14]. The effects of increasing exercise intensity on weight loss when exercise duration is kept constant remain unknown.

High-intensity exercise training may effectively reduce body and abdominal fat [15]. When energy expenditure is held equal, high-intensity exercise is more beneficial for improving body composition and reducing abdominal fat than low-intensity exercise [15, 16]. Furthermore, a randomized controlled trial reported that high-intensity interval training (which resulted in low energy expenditure) was found equally effective in reducing body weight and body fat as was low-intensity endurance exercise training [17]. This finding suggests that a higher exercise intensity may be more effective in improving body composition. However, long-term evidence from randomized controlled trials on the effect of exercise duration with different intensities and energy expenditures on body composition is scant. Furthermore, using a single duration during aerobic exercise training can increase the appetite of obesity people during the recovery period [18]. Moreover, the effects of long-term aerobic exercise training on the appetite and weight loss remain unknown. Therefore, in this pilot randomized controlled trial, we compared the effect of different aerobic exercise intensities and unequal energy expenditures on body composition among sedentary obese college students in Taiwan.

## Material and methods

### Study design and participants

**Fig. 1:**
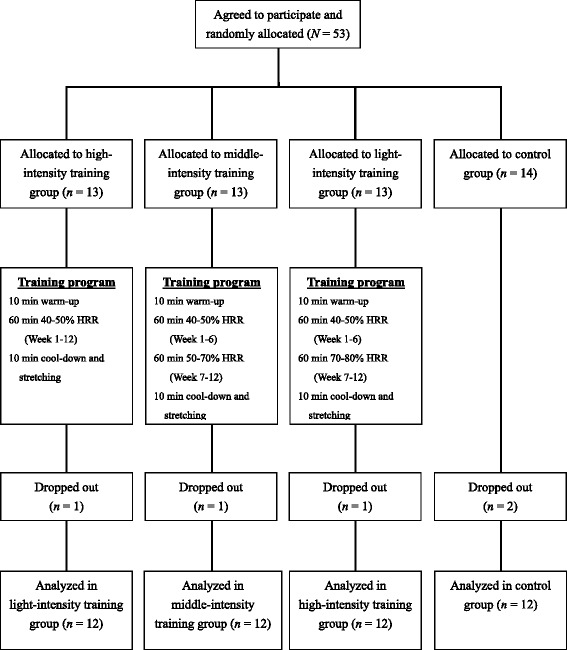
Flow chart of participant enrollment and completion of the 12-week aerobic exercise program

We conducted a 12-week randomized controlled trial. A total of 58 obese sedentary college students (aged 18–26 years) were recruited using advertisements that were posted in the Fitness Center of Chung Hua University in Taiwan. Of these 58 voluntary participants, five did not meet the inclusion criteria; the remaining 53 were randomly assigned into a light-intensity training group (LITG, *n* = 13), middle-intensity training group (MITG, *n* = 13), high-intensity training group (HITG, *n* = 13), and control group (CG, *n* = 14) following initial screening. The inclusion criteria for the recruitment and enrollment of participants were as follows: 1) BMI ≥ 27 kg/m^2^, 2) exercise comprising ≤1 session per week with ≤30 min at moderate-to-vigorous intensity, and 3) a fitness performance level that allowed the completion of the aerobic exercise training in present study. However, five participants dropped out of the study because of ill health, lack of availability, and family commitments. Finally, 48 participants (34 men and 14 women)—with 12 in each group—were evaluated further (Fig. 1). To reduce the influence of other confounding factors, participants with a cardiovascular disease, diabetes, liver dysfunction, renal impairment, a endocrine disorder, a smoking habit, and weight-loss pill consumption were excluded. The experimental protocol was approved by the institutional review board of Taipei Physical Education College, and informed consent was obtained from each participant after fully explaining the study.

### Exercise intervention

The exercise programs, comprising three 60-min sessions weekly for 12 weeks, progressing gradually in intensity were conducted at the Fitness Center of Chung Hua University (Fig. 1). Each session included 10-min warm-up, cool-down, and stretching periods. The LITG performed the aerobic exercise for 40%–50% heart rate reserve (HRR) during weeks 1–12, as determined by using a walking treadmill exercise test. The MITG performed the aerobic exercise for 40%–50% HRR during weeks 1–6, increasing to 50%–70% HRR during weeks 7–12. The HITG performed the aerobic exercise for 40%–50% HRR during weeks 1–6, increasing to 70%–80% HRR during weeks 7–12. Heart rate was monitored continuously on a Polar Accurex monitor (Kempele, Finland) for adjusting workload to achieve the target heart rate. During the 12-week period, all participants were asked not to change their dietary habits; moreover, the CG was asked to not change their exercise habits and maintain normal activity.

### Experimental procedure

Age, sex, smoking habit, current alcohol consumption, and family history of chronic diseases of all participants were recorded. Systolic and diastolic blood pressure after a resting period of at least 5 min was obtained from the right arm through auscultation by using a mercury sphygmomanometer (Baumanometer, Copiague, NY, USA). All participants were instructed to maintain their typical diet and daily physical activity throughout the study period, and compliance with this instruction was assessed through a 24-h diet recall questionnaire and 7-day International Physical Activity Questionnaire administered at the beginning and end of the study. The validity and reliability of these questionnaires have been described elsewhere [19, 20]. Other than diet and physical activity, total energy intake or estimated energy expenditure did not change significantly over the study period (data not shown). Participant demographic data, anthropometric measurements, blood biochemical parameters, and health-related physical fitness components were assessed at baseline and Week 12.

### Health-related physical fitness assessment

The National Physical Fitness Survey conducted by the National Council on Physical Fitness and Sports in Taiwan during 1999–2001 and 2011–2013 included four main components of health-related physical fitness: cardiorespiratory endurance, muscle strength and endurance, flexibility, and body composition. These tests have been used to estimate the health-related physical fitness of people aged 20–65 years and can be performed by research assistants who have attended the official training seminar and passed the certification test on standardized procedures. In present study, body composition and its related outcomes such as cardiorespiratory endurance were measured, which are detailed as follows [21–23]:

- Body composition: Body composition was determined using six anthropometric indices including BMI, WC, hip circumference (HC), waist-to-hip ratio (WHR), waist-to-height ratio (WHtR), and BF%. These measurements were performed after the participants removed their shoes and heavy clothing. The values of body height and weight were recorded in meters to the nearest 0.1 cm and 0.1 kg with a calibrated automatic height and weight scale (model HW686, Taiwan), respectively. BMI was calculated using the following formula: BMI = body weight (in kg)/[height (in m)]^2^. WC was measured to the nearest 0.1 cm by using a flexible steel tape at the level midway between the lowest rib margin and the iliac crest, and HC was measured at the widest level over the greater trochanters. WHR was calculated as WC divided by HC, and WHtR was calculated as WC divided by height. BF%, fat mass, and fat-free mass was determined through bioelectrical impedance analysis by using a Body Composition Analyzer (TANITA Corporation, Japan).
- Cardiorespiratory endurance: Cardiorespiratory endurance was measured using a standardized 3-min step test (step height 35 cm; frequency 24 steps/min). When the exercise was completed, the participants were immediately seated and their heart rates were measured for 1 min, starting within 5 s of the end of the exercise. The sum of the heart rates during the recovery period was compared with the sum of the heart rates during three periods after the test—1–1.5, 2–2.5, and 3–3.5 min [24, 25]. The test was terminated if a subject lost balance, missed the stepping rhythm for three steps, or reported any discomfort during the test [26]. The cardiorespiratory endurance index (CEI) was calculated as CEI = [duration of exercise(s) × 100]/[sum of heart rates during the recovery period/2]. Although maximal exercise testing is a sophisticated measure of cardiorespiratory endurance, generally not feasible or desirable clinically. By contrast, step tests are submaximal exercise tests requiring minimal equipment. Various step tests (different step heights and stepping rates) estimate VO_2_ peak and are reliable and valid measures of cardiopulmonary fitness [27, 28]. The 3-min step test used in our study was adapted from the Harvard Step Test, which assesses cardiorespiratory endurance based on the speed of heart-rate recovery from submaximal exercise. The CEI of the 3-min step test was positively correlated (*r* = 0.50) with VO_2_ peak in healthy Taiwanese adults [29].

The content of the measurements was explained to each participant, and then, they were given a 10-min warm-up and stretching period to achieve their most favorable performance. These measurements were scheduled in the morning before any other exercise. All measurements were performed by the same well-trained investigator. Each measurement was obtained two times, and the average of the two values for each test was used.

### Blood sample analysis

Following a 12-h overnight fasting period, venous blood samples (15 mL) were obtained from an antecubital vein in the sitting position after a 20-min rest between 07:00 a.m. and 09:00 a.m. at baseline and Week 12. The blood was immediately transferred into vacutainer tubes (Becton Dickinson, Rutherford, NJ, USA) containing or not containing 0.1% EDTA as the anticoagulant for estimating the hematological status. Serum or plasma was separated through centrifugation at 2500 rpm for 15 min at 4 °C and then stored at −80 °C until analysis. Hematological entities including fasting glucose, total cholesterol (TC), low-density lipoprotein cholesterol (LDL-C), high-density lipoprotein cholesterol (HDL-C), and triglycerides (TG) were measured using an automated biochemical analyzer. The analytical inter- and intra-assay coefficients of variation obtained in our laboratory were respectively as follows: fasting glucose, 2.4% and 1.6%; TC, 1.3% and 0.7%; LDL-C, 2.2% and 1.6%; HDL-C, 4.5% and 1.6%; and TG, 2.1% and 1.3%.

### Statistical analysis

All analyses were performed using SAS software package (version 9.12, Statistical Analysis System, SAS Institute Inc., Cary, NC, USA). Differences in the participant demographic data, anthropometric measurement, blood biochemical parameters, and health-related physical fitness components between baseline and Week 12 were analyzed using a paired *t* test. Comparisons between the mean values of normally distributed variables between groups of exercise were analyzed using one-way analysis of variance (ANOVA). When a significant *F* value (*p* < 0.05) was noted, Tukey’s post hoc test was performed to determine the differences between the pairs of means. The relationship between changes in various anthropometric indices and physical fitness measurements were examined using Pearson partial correlation coefficients after adjustment for potential confounders. All data are expressed as means ± standard errors (SEs) or frequency percentages (%).We considered a significance level of *p* < 0.05 to reject the null hypothesis. Since this is a pilot study, we did not calculate sample size.

## Results

Baseline characteristics of the four groups including the demographic data, anthropometric measurement, blood biochemical parameters, and health-related physical fitness components did not differ significantly (all *p* > 0.05; Table 1).

**Table 1:** Baseline characteristics of subjects

Characteristic	HITG (*n* = 12)	MITG (*n* = 12)	LITG (*n* = 12)	CG (*n* = 12)	*P*-value
Age (years)	21.75 ± 0.74	20.92 ± 0.38	20.67 ± 0.56	20.83 ± 0.71	0.603
Gender (Male %)	8/4 (66.67)	9/3 (75.00)	8/4 (66.67)	9/3 (75.00)	0.318
Height (cm)	169.17 ± 2.46	170.09 ± 2.84	168.20 ± 2.57	171.40 ± 2.42	0.840
Body weight (kg)	84.38 ± 2.71	92.12 ± 3.89	87.53 ± 4.62	89.83 ± 4.38	0.562
BMI (kg/m^2^)	29.43 ± 0.56	31.78 ± 0.99	30.70 ± 0.99	30.38 ± 0.86	0.305
WC (cm)	97.42 ± 2.08	103.83 ± 2.65	99.38 ± 2.50	98.79 ± 2.86	0.322
HC (cm)	109.96 ± 1.66	115.25 ± 1.97	113.58 ± 2.44	111.83 ± 1.69	0.273
WHR	0.89 ± 0.01	0.90 ± 0.01	0.88 ± 0.02	0.88 ± 0.01	0.680
WHtR	0.58 ± 0.01	0.61 ± 0.02	0.59 ± 0.01	0.58 ± 0.01	0.178
Body fat (%)	34.50 ± 1.78	38.55 ± 2.09	39.89 ± 2.40	33.32 ± 1.07	0.054
Fat mass (kg)	29.04 ± 1.70	35.35 ± 2.24	35.02 ± 2.97	30.03 ± 1.99	0.115
Fat-free mass (kg)	55.34 ± 2.47	56.77 ± 3.34	52.51 ± 3.53	59.80 ± 2.77	0.408
SBP (mmHg)	126.17 ± 2.23	127.75 ± 2.54	119.67 ± 4.24	126.08 ± 1.64	0.203
DBP (mmHg)	75.75 ± 1.87	78.42 ± 2.87	79.25 ± 3.08	83.75 ± 2.59	0.206
Fasting glucose (mg/dL)	89.42 ± 2.53	88.67 ± 2.33	87.83 ± 2.93	86.92 ± 2.77	0.919
TC (mg/dL)	184.67 ± 3.93	185.75 ± 3.67	181.08 ± 4.30	181.50 ± 3.87	0.795
HDL-C (mg/dL)	48.67 ± 2.82	44.42 ± 1.60	47.25 ± 2.67	47.17 ± 1.86	0.619
LDL-C (mg/dL)	116.92 ± 3.06	120.83 ± 3.75	113.67 ± 3.85	111.67 ± 4.12	0.334
TC/HDL-C ratio	3.50 ± 0.16	4.31 ± 0.20	3.83 ± 0.31	3.71 ± 0.25	0.112
TG (mg/dL)	98.50 ± 3.96	114.17 ± 9.94	96.42 ± 11.19	105.67 ± 9.30	0.506

Abbreviations: *BMI* body mass index, *CEI* cardiorespiratory endurance index, *CG* control group, *DBP* diastolic blood pressure, *HC* hip circumference, *HITG* high-intensity training group, *LITG* light-intensity training group, *MITG* moderate-intensity training group, *SBP* systolic blood pressure, *SE* standard error, *TG* triglycerides, *WC* waist circumference, *WHR* waist-to-hip ratio, *WHtR* waist-to-height ratio. Data are expressed as mean ± SE. Groups compared using one-way ANOVA with Tukey’s post hoc comparisons (*P*-values shown)

The changes in the health-related physical fitness components including those in body composition and cardiorespiratory fitness among the four groups between baseline and Week 12 are presented in Table 2. At Week 12, the HITG exhibited significant improvement in body composition in body composition and cardiorespiratory fitness measurements (*p* ≤ 0.05). Similarly, the body composition and cardiorespiratory fitness measurements significantly improved in the MITG and LITG; in addition, no differences in fat-free mass were observed in the MITG and LITG and no significant differences in HC, fat-free mass, and sit-and-reach test in the LITG. No significant changes were observed in any of these outcome measurements in the CG.

**Table 2:** The comparison of body composition and cardiorespiratory fitness measurements following 12-week walking programs

Variables	HITG (*n* = 12)			MITG (*n* = 12)			LITG (*n* = 12)			CG (*n* = 12)		
	Baseline	Week 12	*P*-value	Baseline	Week 12	*P*-value	Baseline	Week 12	*P*-value	Baseline	Week 12	*P*-value
Body weight (kg)	84.38 ± 2.71	77.66 ± 2.33	< 0.0001	92.12 ± 3.89	87.15 ± 3.51	< 0.0001	87.53 ± 4.62	84.60 ± 4.50	< 0.0001	89.83 ± 4.38	90.57 ± 4.40	0.138
BMI (kg/m^2^)	29.43 ± 0.56	27.08 ± 0.39	< 0.0001	31.78 ± 0.99	30.08 ± 0.91	< 0.0001	30.70 ± 0.99	29.67 ± 0.94	< 0.0001	30.38 ± 0.86	30.64 ± 0.88	0.127
WC (cm)	97.42 ± 2.08	88.04 ± 1.88	< 0.0001	103.83 ± 2.65	95.92 ± 2.18	< 0.0001	99.38 ± 2.50	94.12 ± 2.36	< 0.0001	98.79 ± 2.86	99.25 ± 2.81	0.298
HC (cm)	109.96 ± 1.66	109.00 ± 1.77	0.004	115.25 ± 1.97	113.92 ± 1.66	0.034	113.58 ± 2.44	112.21 ± 2.00	0.054	111.83 ± 1.69	112.67 ± 1.72	0.277
WHR	0.89 ± 0.01	0.81 ± 0.01	< 0.0001	0.90 ± 0.01	0.84 ± 0.01	< 0.0001	0.88 ± 0.02	0.84 ± 0.02	< 0.0001	0.88 ± 0.01	0.88 ± 0.01	0.560
WHtR	0.58 ± 0.01	0.52 ± 0.01	< 0.0001	0.61 ± 0.02	0.57 ± 0.01	< 0.0001	0.59 ± 0.01	0.56 ± 0.01	< 0.0001	0.58 ± 0.01	0.58 ± 0.01	0.282
Percent body fat (%)	34.50 ± 1.78	30.53 ± 1.60	< 0.0001	38.55 ± 2.09	35.64 ± 1.95	< 0.0001	39.89 ± 2.40	38.19 ± 2.37	< 0.0001	33.32 ± 1.07	33.54 ± 1.20	0.361
Fat mass (kg)	29.04 ± 1.70	23.60 ± 1.29	< 0.0001	35.35 ± 2.24	30.99 ± 2.05	< 0.0001	35.02 ± 2.97	32.40 ± 2.79	< 0.0001	30.03 ± 1.99	30.48 ± 2.07	0.054
Fat-free mass (kg)	55.34 ± 2.47	54.06 ± 2.28	0.010	56.77 ± 3.34	56.16 ± 2.94	0.238	52.51 ± 3.53	52.20 ± 3.48	0.067	59.80 ± 2.77	60.09 ± 2.78	0.500
CEI	56.82 ± 3.12	64.24 ± 3.26	< 0.0001	52.16 ± 1.24	57.04 ± 1.27	< 0.0001	51.50 ± 1.93	54.69 ± 1.85	< 0.0001	55.30 ± 1.80	54.78 ± 1.71	0.122

Abbreviations: *BMI* body mass index, *CEI* cardiorespiratory endurance index, *CG* control group, *HC* hip circumference, *HITG* high-intensity training group, *LITG* light-intensity training group, *MITG* moderate-intensity training group, *SE* standard error, *WC* waist circumference, *WHR* waist-to-hip ratio, *WHtR* waist-to-height ratio. Data are expressed as mean ± SE. Significantly different between baseline and week 12 by paired *t*-test (*P* < 0.05)

Table 3 also presents the differences in the changes in health-related physical fitness components among the four groups. ANOVA indicated significant means group effects for changes in the body weight, BMI, WC, HC, WHR, WHtR, %BF, fat mass, and CEI (all *p* < 0.05); however, the changes in fat-free mass did not have significant differences in the group effects. Furthermore, post hoc comparisons revealed that the changes in body weight, WC, WHR, WHtR, fat mass, and CEI were significantly higher in the HITG and MITG compared with the LITG and CG; changes in the HITG and MITG did not differ significantly, whereas those in the LITG and CG did. In addition, the changes in BMI and BF% significantly differed among the four groups.

**Table 3:** Changes in body composition and cardiorespiratory fitness measurements following 12-week walking programs

Variables	HITG (*n* = 12)	MITG (*n* = 12)	LITG (*n* = 12)	CG (*n* = 12)	*P*-value	Tukey’s post hoc test
Body weight (kg)	−6.73 ± 0.67	−4.97 ± 0.46	−2.93 ± 0.19	0.74 ± 0.46	< 0.0001	HITG, MITG > LITG > CG
BMI (kg/m^2^)	−2.34 ± 0.24	−1.70 ± 0.13	−1.04 ± 0.06	0.26 ± 0.16	< 0.0001	HITG > MITG > LITG > CG
WC (cm)	−9.38 ± 0.74	−7.92 ± 0.69	−5.25 ± 0.23	0.46 ± 0.42	< 0.0001	HITG, MITG > LITG > CG
HC (cm)	−0.96 ± 0.26	−1.33 ± 0.55	−1.38 ± 0.64	0.83 ± 0.73	0.028	MITG, LITG > CG
WHR	−0.08 ± 0.01	−0.06 ± 0.01	−0.04 ± 0.004	−0.002 ± 0.004	< 0.0001	HITG, MITG > LITG > CG
WHtR	−0.06 ± 0.004	−0.05 ± 0.004	−0.03 ± 0.001	0.003 ± 0.002	< 0.0001	HITG, MITG > LITG > CG
Percent body fat (%)	−3.97 ± 0.28	−2.91 ± 0.25	−1.70 ± 0.14	0.23 ± 0.24	< 0.0001	HITG > MITG > LITG > CG
Fat mass (kg)	−5.44 ± 0.49	−4.35 ± 0.23	−2.62 ± 0.22	0.45 ± 0.21	< 0.0001	HITG, MITG > LITG > CG
Fat-free mass (kg)	−1.29 ± 0.42	−0.61 ± 0.49	−0.31 ± 0.15	0.29 ± 0.42	0.050	HITG > CG
CEI	7.42 ± 0.34	4.88 ± 0.28	3.19 ± 0.16	−0.52 ± 0.31	< 0.0001	HITG, MITG > LITG > CG

Abbreviations: *BMI* body mass index, *CEI* cardiorespiratory endurance index, *CG* control group, *HC* hip circumference, *HITG* high-intensity training group, *LITG* light-intensity training group, *MITG* moderate-intensity training group, *SE* standard error, *WC* waist circumference, *WHR* waist-to-hip ratio, *WHtR* waist-to-height ratio. Data are expressed as mean ± SE. Groups compared using one-way ANOVA with Tukey’s post hoc comparisons (*P* < 0.05)

Moreover, pooled partial Pearson correlation analyses (*n* = 48) were conducted to examine the relationship among changes in cardiorespiratory fitness and body composition after adjusting for age and sex (Table 4). The changes in CEI were negatively associated with changes in body weight, BMI, WC, HC, WHR, WHtR, %BF, fat mass, and fat-free mass (*p* < 0.05).

**Table 4:** Correlates of changes in body composition measurements with change in cardiorespiratory fitness

Variables	△ CEI	
	*r*	*P*-value
△ Body weight (kg)	−0.857	< 0.0001
△ BMI (kg/m^2^)	−0.848	< 0.0001
△ WC (cm)	−0845	< 0.0001
△ HC (cm)	−0.345	0.019
△ WHR	−0.798	< 0.0001
△ WHtR	−0.841	< 0.0001
△ Percent body fat (%)	−0.843	< 0.0001
△ Fat mass (kg)	−0.871	< 0.0001
△ Fat-free mass (kg)	−0.442	0.002

Abbreviations: *BMI* body mass index, *CEI* cardiorespiratory endurance index, *CG* control group, *HC* hip circumference, *HITG* high-intensity training group, *LITG* light-intensity training group, *MITG* moderate-intensity training group, *WC* waist circumference, *WHR* waist-to-hip ratio, *WHtR* waist-to-height ratio. △, change in. Data are expressed as Pearson correlation coefficients after adjusting for age and gender (*P* < 0.05). Data derived from pooled groups (*N* = 48)

## Discussion

In this randomized controlled trial, we examined the effect of aerobic exercise at three intensities on body weight and body fat of obese individuals. After a 12-week exercise intervention, higher intensity exercise training (HITG and MITG) led to significantly more changes in body composition. Body weight, WC, WHR, and WHtR were significantly improved in the HITG and MITG compared with those in the LITG and CG. Furthermore, low-intensity exercise intervention without diet control also improved body composition. This study emphasizes that regular exercise training is a major factor responsible for improving body composition and preventing abdominal obesity-related chronic disease in overweight and obese people.

Our results are consistent with those of previous studies: high-intensity training is more effective in decreasing BF% [15]. The higher exercise intensity and energy expenditure may be involved in improving body composition [30, 31]. Compared with low-intensity exercise, high-intensity exercise may reduce body weight and body fat significantly, when the energy expenditure is equal [15, 16]. The possible explanations for the effect of exercise intensity on body composition control are that high intensity exercise can increase catecholamine and growth hormone release [32, 33], postexercise oxygen consumption [34, 35], and lipoprotein lipase activity [36]. By contrast, higher energy expenditure during exercise can cause greater body fat loss [30, 31]. Taken together, higher exercise intensity may result in significantly higher long-term reduction in body fat compared with lower exercise intensity.

Previous studies examining the effect of exercise training on appetite perceptions during postexercise recovery have reported inconsistent results [37–42]. The present study demonstrated that aerobic exercise training may not affect appetite perceptions when the energy intake is not controlled. This finding is consistent with recent studies showing that long-term exercise training may not affect energy intake and reduce the benefit of weight loss [37, 39]. Studies have also suggested that high-intensity exercise results in reducing energy intake after exercise [41, 42]. No association between energy expenditure during exercise and energy intake or appetite perceptions was reported previously [37, 40, 41]. In present study, no such association was observed over the study period: high-intensity exercise training with more energy expenditure effectively improved body composition. Our findings indicate that high-intensity exercise training with higher energy expenditure is more effective in improving body composition than low-intensity exercise training, even when the diet is not controlled.

The differences in exercise intensities resulted in significant changes in body weight, WC, WHR, and WHtR (HITG and MITG > LITG > CG) among our participants. Thus, intensity may be crucial for reducing abdominal fat and sequentially reducing WC, WHR, and WHtR. Higher exercise intensity, but not high energy expenditure, may significantly reduce whole body fat, abdominal fat, subcutaneous abdominal fat, and abdominal visceral fat compared with lower exercise intensity [15, 16]. By contrast, energy expenditure during aerobic exercise training is only associated with visceral fat and not subcutaneous or abdominal fat [31, 43]. Although not statistically significant, the values of reduction in WC, WHR, and WHtR was higher in the HITG than in the MITG. Thus, exercise intensity is crucial for modulating abdominal fat. Furthermore, the additional benefit of abdominal fat reduction may be counteracted if the exercise intensity is higher than 50%–80% HRR.

The results of present study implicate that low-intensity exercise can also significantly reduce body weight and body fat: body weight (−2.9 ± 0.2 kg, *p* < 0.001) and body fat (−1.7% ± 0.1%) decreased significantly in the LITG. Exercise training for 150–250 min per week effectively reduces body weight. Exercise at the similar duration and intensity can also reduce body weight and body fat [44]. These findings are consistent with those of our study, suggesting that three sessions of light-intensity training for 60 min per day effectively reduces body weight and body fat in sedentary obese people.

The main limitation of the present study was that we only collected data before and after the exercise intervention. Hence, the speed of weight loss during the study is unknown. Additionally, the exercise intervention was only 12 weeks in duration, and therefore was not suitable for follow-up examinations. Future research must investigate the speed of weight loss (and fluctuations therein) by using a longer exercise intervention period.

## Conclusion

In conclusion, higher exercise intensity and energy expenditure can significantly reduce body weight, body fat, WC, WHR, and WHtR. Light-intensity exercise training can also significantly reduce body weight and body fat. Here, a 12-week aerobic exercise program significantly modulated the anthropometric indices in obese college students, even when the diet was not controlled. Accordingly, when prescribing an aerobic exercise training program for individuals with obesity related issues, a clinical practitioner should take the intensity of the training into consideration. A high intensity aerobic exercise training program should be prescribed by a clinical practitioner for an obese individual when the individual’s exercise capacity is high enough to complete the program.

## Abbreviations

ACSM: American College of Sports Medicine
ANOVA: Analysis of variance
BF%: Body fat percentage
BMI: Body mass index
CEI: Cardiorespiratory endurance index
CG: Control group
HC: Hip circumference
HDL-C: High-density lipoprotein cholesterol
HITG: High-intensity training group
HRR: Heart rate reserve
IPAQ: International Physical Activity Questionnaire
LDL-C: Low-density lipoprotein cholesterol
LITG: Light-intensity training group
MITG: Middle-intensity training group
MOHW: Ministry of Health and Welfare
SE: Standard error
TC: Total cholesterol
TG: Triglycerides
VO_2_peak: Peak oxygen consumption
WC: Waist circumference
WHO: World Health Organization
WHR: Waist-to-hip ratio
WHtR: Waist-to-height ratio
